# Impact of differential and time-dependent autophagy activation on therapeutic efficacy in a model of Huntington disease

**DOI:** 10.1080/15548627.2020.1760014

**Published:** 2020-05-06

**Authors:** Per Ludvik Brattås, Bob A. Hersbach, Sofia Madsen, Rebecca Petri, Johan Jakobsson, Karolina Pircs

**Affiliations:** Laboratory of Molecular Neurogenetics, Department of Experimental Medical Science, Wallenberg Neuroscience Center and Lund Stem Cell Center, Lund University, Lund, Sweden

**Keywords:** Adeno-associated viral vectors, autophagy, BECN1/beclin-1, Huntington disease, macroautophagy, neurodegeneration, TFEB

## Abstract

Activation of macroautophagy/autophagy, a key mechanism involved in the degradation and removal of aggregated proteins, can successfully reverse Huntington disease phenotypes in various model systems. How neuronal autophagy impairments need to be considered in Huntington disease progression to achieve a therapeutic effect is currently not known. In this study, we used a mouse model of HTT (huntingtin) protein aggregation to investigate how different methods and timing of autophagy activation influence the efficacy of autophagy-activating treatment *in vivo*. We found that overexpression of human *TFEB*, a master regulator of autophagy, did not decrease mutant HTT aggregation. On the other hand, *Becn1* overexpression, an autophagic regulator that plays a key role in autophagosome formation, partially cleared mutant HTT aggregates and restored neuronal pathology, but only when administered early in the disease progression. When *Becn1* was administered at a later stage, when prominent mutant HTT accumulation and autophagy impairments have occurred, *Becn1* overexpression did not rescue the mutant HTT-associated phenotypes. Together, these results demonstrate that the targets used to activate autophagy, as well as the timing of autophagy activation, are crucial for achieving efficient therapeutic effects.**Abbreviations**: AAV: adeno-associated viral vectors; ACTB: actin beta; BECN1: beclin 1, autophagy related; DAPI: 4ʹ,6-diamidino-2-phenylindole; GO: gene ontology; HD: Huntington disease; HTT: huntingtin; ICQ: Li’s intensity correlation quotient; IHC: immunohistochemistry; LAMP1: lysosomal-associated membrane protein 1; MAP1LC3B/LC3B: microtubule-associated protein 1 light chain 3 beta; mHTT: mutant huntingtin; PCA: principal component analysis; PPP1R1B/DARPP-32: protein phosphatase 1 regulatory inhibitor subunit 1B; SQSTM1: sequestosome 1; TFEB: transcription factor EB; WB: western blot; WT: wild-type.

## Introduction

Huntington disease (HD) is a neurodegenerative disorder that is inherited in an autosomal dominant fashion and exhibits, as part of its core pathology, a predominant loss of cortical and striatal medium spiny neurons. HD is a fatal disorder characterized by involuntary movements coupled with cognitive impairment and emotional lability, with no efficient treatment currently available. At a molecular level, the disease is caused by an expansion of CAG trinucleotide repeats in the first exon of the *HTT* (huntingtin) gene, which results in an elongated polyQ stretch in the mutated protein [1]. Consequently, mutant HTT (mHTT) is more prone to form oligomers and eventually aggregates into the inclusions that characterize the disease histologically [1,2]. There is a clear negative correlation between the number of CAG repeats and the disease onset, whereas the age-dependent neuronal loss and the severity of symptoms positively correlate with the accumulation of mHTT into protein aggregates [3,4].

Several studies have demonstrated that the presence of mHTT interrupts macroautophagy (hereafter referred to as autophagy), contributing to the impaired clearance of aggregated proteins [5–8]. Autophagy is a lysosomal degradation pathway that is present at a basal level in all eukaryotic cells and ensures steady-state homeostasis. It has an essential role in normal cytoplasmic turnover and eliminating damaged, dysfunctional, and unused cellular components. Autophagy is characterized by the formation of a phagophore, a double-membraned compartment that first engulfs the cytoplasmic cargo; the phagophore matures into an autophagosome, which then fuses with a lysosome, forming an autolysosome, where the cargo is eventually degraded. Autophagy is a key mechanism involved in the degradation and removal of aggregated proteins, and inhibition of constitutively active autophagy has been shown to cause neurodegeneration in the brain [9,10].

In the models of HD, impairments in autophagy result in an increased number of autophagosomes, many of which are empty due to a cargo recognition failure [6]. Moreover, mHTT disrupts vesicle trafficking, autophagosome-lysosome fusion and dynamics [5,8,11]. These specific defects in the autophagic machinery can lead to a negative feedback loop, where mHTT aggregation leads to further dysregulation of autophagy, causing increased mHTT accumulation and neurotoxicity. Overall, these data demonstrate that expression of m*HTT* results in a distinct and complex impairment of autophagy in HD. However, very little is known about how such impairments need to be considered when determining how and when to boost autophagy during the disease process in order to achieve optimal therapeutic effects.

Given the alterations of autophagy in animal models of HD, as well as in postmortem tissue from patients, therapeutic strategies that boost autophagy have been tested in pre-clinical disease models, as well as in a phase-I clinical trial [12,13]. Indeed, activation of autophagy using pharmacological or genetic manipulation successfully reverses HD-associated phenotypes in both fly and mouse models [5,14–19]. For example, TFEB (transcription factor EB), a master regulator of autophagy, has previously been used as a potential autophagy-lysosomal pathway inducer, which positively modulates autophagy through autophagosome formation and autophagosome-lysosome fusion [20–22]. *TFEB* activation has successfully ameliorated toxic protein aggregate levels in mouse models of different proteinopathies, such as Alzheimer disease, Parkinson disease, and HD [22–25].

Another gene of interest in this context is *BECN1* (beclin 1), the mammalian ortholog of *VPS30/ATG6* and an autophagic regulator that plays a key role in autophagosome formation. Overexpression of *BECN1* delays the onset and slows the progress of HD in both cell and mouse models by inducing autophagy [5,26]. Expression of m*HTT* has also been shown to lower BECN1 levels and decrease autophagosome biogenesis, which is associated with enhanced mHTT aggregation, and the expression of BECN1 in the brain of human HD patients declines with age, supporting the importance of *BECN1* in HD pathology [26–29]. Together, these studies suggest that both *TFEB* and *BECN1* may serve as useful targets for the treatment of HD through stimulation of autophagy.

In this study, we used an adeno-associated viral vector (AAV)-based mouse model of HD that allows a time-dependent investigation of how autophagy is affected by mHTT aggregation in striatal neurons *in vivo* [5]. We found that overexpression of the human m*HTT* initially resulted in activated autophagy. However, this activation was rapidly replaced by a robust impairment in autophagy that correlated with the presence of mHTT aggregates. In this dynamic model, we investigated how different modes and timing of autophagy activation influences the efficacy of mHTT clearance. We found that *TFEB* overexpression did not change the amount of mHTT-aggregates, whereas *Becn1* overexpression cleared mHTT aggregates, but only when administered early in the disease course. When *Becn1* was overexpressed at a later stage, when prominent mHTT accumulation and clear autophagy impairments were present, autophagy induction did not rescue the m*HTT*-associated phenotypes. Together, our results demonstrate that the choice of the therapeutic target, as well as early delivery, are crucial parameters in order to reverse the m*HTT*-associated phenotypes.

## Results

### An AAV-based model of HD allows for the investigation of early disease progression

**Figure 1.**
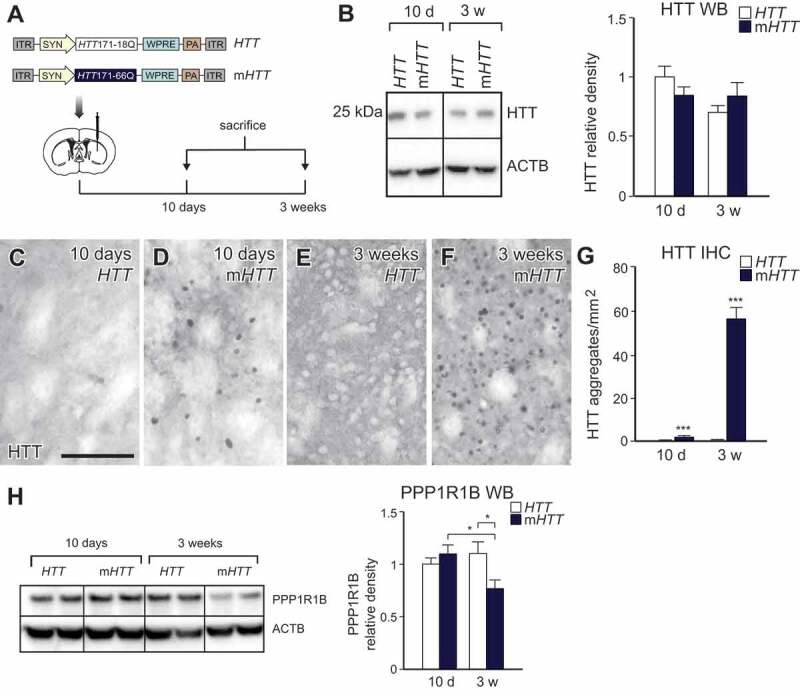
An AAV-based model of HD allows the investigation of disease progression. (A) Diagrams of the AAV-m*HTT* and AAV-*HTT* vectors and the experimental workflow. (B) Expression levels of truncated human HTT and human mHTT protein were present both in the AAV-*HTT-* and AAV-m*HTT*-injected animals after 10 d and 3 weeks. There was no significant difference between any groups at any time-points. (n = 9; 4–4 animals in each group). (C-G) mHTT aggregates accumulate 10 d after AAV-m*HTT* injection. The amount increases 6-fold by 3 weeks. AAV-*HTT*-injected animals did not develop any aggregates. (n = 20; 4–4 animals in each group). (H) Decreased expression levels of PPP1R1B were present in the AAV-m*HTT*-injected animals after 3 weeks. There was no significant difference after 10 d between AAV-*HTT-* and m*HTT*-injected animals. (n = 11 for 10 d and n = 9 for 3 weeks; 4–4 animals in each group). ***p < 0.001; **p < 0.01; *p < 0.05; two-tailed two-sample T-tests in G or one-way ANOVA test were used based on normal distribution defined by D’Agostino-Pearson omnibus normality test in B and H. All data are shown as mean ± SEM. WB values were normalized to 10 d AAV-*HTT* expression levels and corrected to ACTB (actin beta) values. Scale bar: 50 μm

To model time-dependent impairment of autophagy upon m*HTT* expression in striatal neurons, we used AAV5 vectors expressing the exon 1 of wild-type (WT), human HTT (*HTT*, 18 polyQ repeats) and mutant human HTT (m*HTT*, 66 polyQ repeats) under the control of the neuron-specific *Syn1* (synapsin 1) promoter (Figure 1A) [5]. AAV-*HTT* and AAV-m*HTT* were injected into the striatum of mice that were sacrificed at either 10 d or 3 weeks post-injection, time-points selected to represent early- and late-stage of the disease progression and autophagy impairment (Figure 1A). We first performed a western blot (WB) on the striatal tissue by staining with an EM48-antibody (detects both WT and mHTT) and found that WT HTT and mHTT were overexpressed in striatal tissue at similar levels at both time-points, confirming the efficient AAV-mediated overexpression, as well as similar dosage of the transgene expression in the two experimental groups (Figure 1B).

To confirm that the expression of m*HTT* resulted in the formation of protein aggregates, we performed immunohistochemistry (IHC) using the EM48-antibody. Mice injected with AAV-*HTT* did not develop HTT aggregates, while mice injected with AAV-m*HTT* showed a small number of intracellular mHTT aggregates at 10 d post-injection, which increased drastically after 3 weeks (Figure 1C–Figure 1G). In addition, we also measured the levels of PPP1R1B/DARPP-32 (protein phosphatase 1 regulatory inhibitor subunit 1B), a component of the dopamine signaling pathway used as an indicator of pathological progression in HD [30,31], and found a reduction in PPP1R1B levels only in AAV-m*HTT*-injected mice using WB after 3 weeks but not after 10 d post-injection (Figure 1H). Thus, these data demonstrate that this AAV-based strategy is a suitable *in vivo* model to study early progressive alterations in neuronal function during mHTT protein aggregation [5].

### Altered autophagy following protein aggregation in striatal neurons

We next analyzed how mHTT aggregation affected autophagy at 10 d (early time-point) and 3 weeks (late time-point) post-injection. To this end, we evaluated autophagy with WB and IHC using several markers of autophagy as a read-out, while also performing transcriptome analysis with next-generation sequencing in striatal tissue from mice injected with AAV-m*HTT* and AAV-*HTT*.

**Figure 2.**
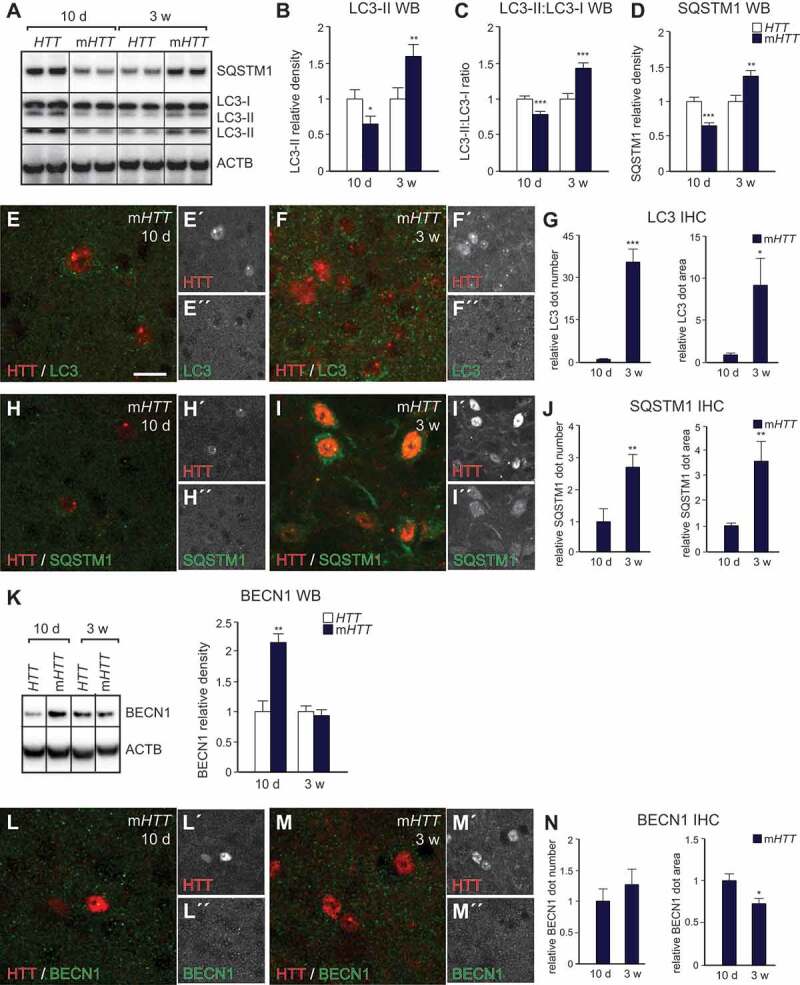
m*HTT* overexpression alters autophagy on a protein level in striatal neurons. (A-D) Decreased expression levels of the autophagic markers SQSTM1, LC3-II coupled with decreased LC3-II:LC3-I ratio were detected at 10 d after AAV-m*HTT* injections. At 3 weeks post-injection, the opposite effects were detected in the AAV-m*HTT*-injected animals. (n = 11 for SQSTM1; n = 8 for LC3; 4–4 animals in each group). (E-J) Both the number and size of LC3 and SQSTM1 puncta significantly increased in the 3-week AAV-m*HTT*-injected animals compared to 10 d. (n = 15 for LC3; n = 15 for 10 d and 12 for 3 weeks for SQSTM1; 3–3 animals in each group). (K) Accumulation of BECN1 was present after 10 d, but no change was seen after 3 weeks in the AAV-m*HTT*-injected mice. (n = 7; 4–4 animals in each group). (L-N) BECN1 dot number remained the same, while the area of the dots significantly decreased 3 weeks after AAV-m*HTT* injection compared to 10 d. (n = 12; 3–3 animals in each group). ***p < 0.001; **p < 0.01; *p < 0.05; two-tailed two-sample T-tests were used. All data are shown as mean ± SEM. WB values were normalized to 10 d or 3 weeks AAV-*HTT* expression levels and corrected to ACTB values. IHC values were normalized to the 10 d AAV-m*HTT* dot number or area. Scale bar: 25 μm

At the early time-point, we detected decreased protein levels of the autophagosomal marker MAP1LC3B-II/LC3-II (microtubule-associated protein 1 light chain 3 beta, lipidated) in the AAV-m*HTT*-injected animals compared to the AAV-*HTT* (Figure 2A,Figure 2B,Figure 2E–Figure 2G). We also measured the ratio between the membrane-bound autophagosome-associated LC3‑II and cytoplasmic LC3‑I by immunoblot and found a significant decrease of the membrane-bound LC3-II after m*HTT* injection (Figure 2A,Figure 2C). Additionally, we detected decreased SQSTM1 (sequestosome 1), coupled with an increased lysosomal/endosomal LAMP1 (lysosomal-associated membrane protein 1) level (Figure 2A,Figure 2D,Figure 2H–Figure 2J, and S1A-F). SQSTM1 is selectively degraded by autophagy and therefore, the level of SQSTM1 negatively correlates with autophagy [32]. These data suggested an increased autophagosomal turnover 10 d after AAV-m*HTT* injection. Additionally, at this early time-point we detected a 2-fold increase in the protein level of BECN1, a key component involved in autophagy induction and autophagosome formation, which further demonstrated increased autophagy upon AAV-m*HTT* overexpression (Figure 2K–Figure 2N). We investigated the protein expression level of TFEB, a key regulator of the autophagy-lysosomal pathway, and found no difference between AAV-*HTT* and m*HTT*-injected animals (Fig. S1G-J). Altogether, these data demonstrated activation of autophagy due to AAV-m*HTT* injection compared to the AAV-*HTT*-injected animals at an early time-point.

At the late time-point, when prominent mHTT aggregation was present, we found an opposite effect on autophagy upon AAV-m*HTT* injection. Levels of SQSTM1, LC3-II, LC3-II:LC3-I ratio, and LAMP1 increased 3 weeks after AAV-m*HTT* injection compared to the AAV-*HTT*-injected animals (Figure 2A–Figure 2J and S1A-F). Elevated levels of these autophagy markers in the AAV-m*HTT*-injected animals indicated impairment in either the autophagosome-lysosome fusion or in the lysosome-mediated proteolysis. Additionally, we found that BECN1 and TFEB levels remained unchanged at 3 weeks between AAV-m*HTT* and AAV-*HTT*-injected animals (Figure 2K–Figure 2N and S1G-J). The reduction of BECN1 levels in the AAV-m*HTT*-injected animals compared to the early time-point indicate that there was no longer an induction of autophagy upon AAV-m*HTT* overexpression, and rather the opposite, that autophagy was impaired at a later time-point.

When directly comparing AAV-m*HTT*-injected animals in the early and late time-points using IHC, we found a significant increase in both the number and size of LC3 and SQSTM1 puncta in the late AAV-m*HTT* animals compared to early AAV-m*HTT*-injected animals (Figure 2E–Figure 2J). While the number of BECN1-, LAMP1-, and TFEB-positive dots were not changed between the early and late AAV-m*HTT*-injected animals, BECN1 and LAMP1 puncta size were significantly decreased after 3 weeks (Figure 2L–Figure 2N, S1D-F, and S1H-J). Altogether, these data further suggest an impaired autophagosome-lysosome fusion or inhibition in the lysosome-mediated proteolysis in the late time-point AAV-m*HTT*-injected animals.

In summary, these results indicated that while autophagy was activated at an early time-point, autophagy was impaired 3 weeks after m*HTT* injection when prominent mHTT aggregation is present. Furthermore, our analysis using several different autophagy markers demonstrates that the impairment is characterized by a late autophagy-lysosomal defect at 3 weeks after AAV-m*HTT* injection.

**Figure 3.**
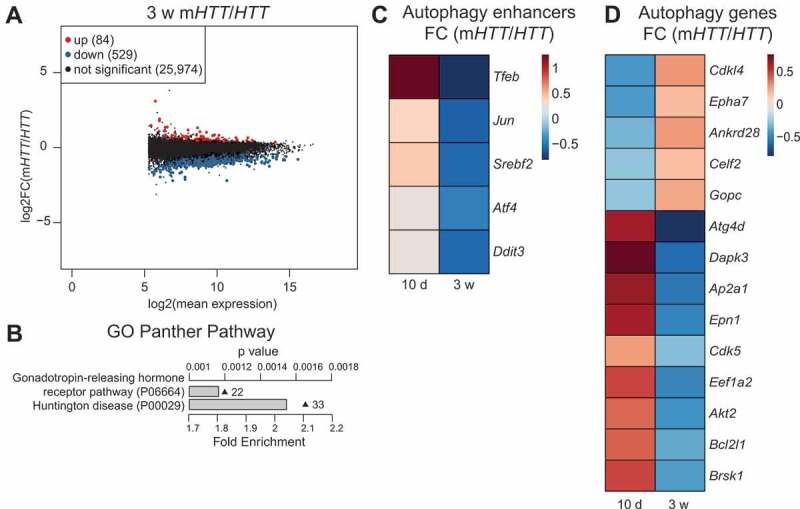
m*HTT* overexpression alters autophagy on a transcriptional level in striatal neurons. (A) Majority of differentially expressed genes 3 weeks after AAV-m*HTT* injection are significantly decreased. (n = 3 for *HTT* and n = 2 for m*HTT* animals/group at the 3-week time-points). (B) Panther pathway GO analysis top hit shows involvement in HD for the differentially downregulated genes after AAV-m*HTT* injection at 3 weeks. Grey bars show fold enrichment. Triangle points show p-value. Numbers indicate the number of significantly downregulated genes for each GO-term. (n = 3 for *HTT* and n = 2 for m*HTT* animals/group at the 3-week time-points). (C) Heatmap of transcription factors enhancing autophagy shows an increased expression at 10 d and a decrease at 3 weeks post-injection. (n = 2 for *HTT* and n = 3 for m*HTT* animals/group at 10 d and n = 3 for *HTT* and n = 2 for m*HTT* animals/group at 3-week time-points). (D) Heatmap of significantly differentially expressed genes involved in autophagy both at 10 d and 3 weeks show opposite expression levels at 10 d and 3 weeks post-injection. (n = 2 for *HTT* and n = 3 for m*HTT* animals/group at 10 d and n = 3 for *HTT* and n = 2 for m*HTT* animals/group at 3-week time-points). *p < 0.05; Benjamini-Hochberg method was used as the cutoff for significance in Wald test

It is well established that m*HTT* induces transcriptional alterations, which may play a part in autophagy dysregulation [5,22,33]. To investigate transcriptional changes in our model, we performed RNA-sequencing on freshly dissected striatal tissue from AAV-*HTT* or m*HTT*-injected animals after 10 d and 3 weeks. Principal component analysis (PCA) showed a clear linear separation between animals sacrificed after 10 d and 3 weeks, but only between AAV-*HTT* and m*HTT*-injected animals at 3 weeks, indicating the progressive nature of our model (Fig. S2A and S2B). Strikingly, in the 3-week AAV-m*HTT* group, almost all significantly differentially expressed genes were downregulated when compared to both 10 d AAV-m*HTT* or 3 weeks AAV-*HTT*-injected animals (Figure 3A and S2C). GO term analysis of genes differentially expressed at this late time-point showed significant enrichment for genes involved in HD, along with pathways known to be altered in HD, such as nervous system development and oxidative phosphorylation (Figure 3B and S2D).

When autophagy was activated 10 d after m*HTT* injection, we found increased transcription levels of several transcription factors known to enhance autophagy (Figure 3C). In contrast, at 3 weeks after AAV-m*HTT* injection, when autophagy was reduced, we found decreased levels of these transcription factors, further indicating an autophagy alteration at a later stage (Figure 3C). Changes in the transcription factor levels were also associated with large-scale transcriptional changes in many genes implicated in cargo recruitment, vesicle regulation, and lysosomal functions (Figure 3D and S2E). However, it is worth noting that while *Tfeb* was the most differentially expressed autophagy enhancer in the early and late AAV-m*HTT*-injected animals, there was no change of TFEB protein levels between any of the groups, indicating a post-transcriptional control of TFEB levels (Figure 3C and Fig. S1G-J). Cumulatively, these results show that a transcriptional response contributes to the induction of autophagy found at 10 d post-AAV-m*HTT* injection and that there is a component of transcriptional dysregulation that contributes to the decreased autophagy found at later time-points.

### Induction of autophagy by Tfeb overexpression does not reverse HD-like phenotypes

**Figure 4.**
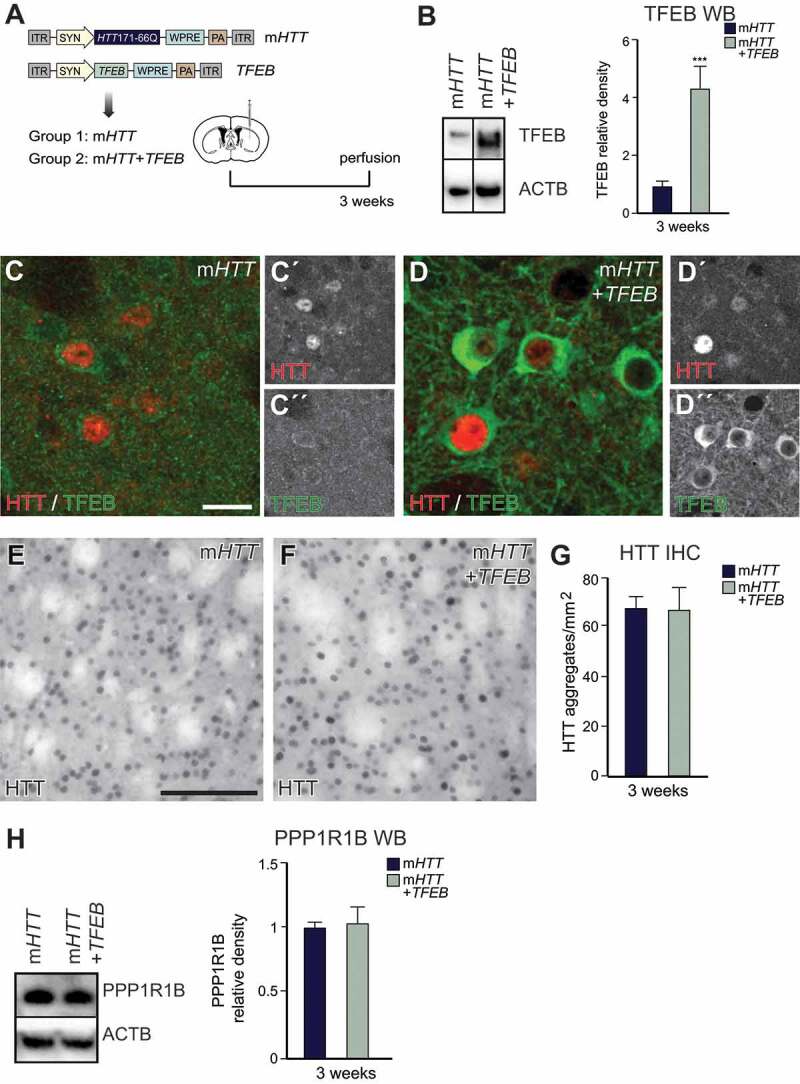
Autophagy induction by human *TFEB* overexpression does not reverse HD-like phenotypes I. (A) Experimental workflow summarizing the co-delivery of AAV-m*HTT* and AAV-*TFEB*. (B) WB analysis verifying successful human *TFEB* overexpression after the co-injection of AAV-m*HTT* and *TFEB*. (n = 6 for m*HTT* and 5 for *TFEB* co-injected animals; 4–4 animals in each group). (C and D) IHC analysis verifying successful *TFEB* overexpression after the co-injection of AAV-m*HTT* + *TFEB* using the TFEB antibody. (E–G) Co-injection of AAV-*TFEB* and AAV-m*HTT* together did not decrease the aggregation of mHTT. (n = 20; 4–4 animals in each group). (H) PPP1R1B protein levels were similar in the AAV-m*HTT* + *TFEB* co-injected animals compared to AAV-m*HTT* alone measured with WB. (n = 4; 4–4 animals in each group). ***p < 0.001; **p < 0.01; *p < 0.05; two-tailed two-sample T-tests were used. All data are shown as mean ± SEM. WB values were normalized to AAV-m*HTT* expression levels and corrected to ACTB values. Scale bars: 20 μm in C for C and D; 50 μm in E for E and F

As mentioned above, a number of previous studies in cell culture and mouse models of HD have indicated that *TFEB* overexpression reduces HTT protein aggregation [21,22,34]. Thus, *TFEB* may be a useful target to activate autophagy for the treatment of HD. We used an AAV5-*TFEB* overexpression vector to co-inject human *TFEB* together with AAV-m*HTT* in the mouse striatum (Figure 4A) [23]. We verified efficient TFEB protein overexpression by WB and IHC 3 weeks after co-injection with AAV-m*HTT* and detected a clear increase of TFEB (Figure 4B–Figure 4D). However, we did not detect any changes in the amount of mHTT aggregates in the co-injected animals (Figure 4E–Figure 4G and S3A). We also did not find any changes in PPP1R1B levels (Figure 4H).

**Figure 5.**
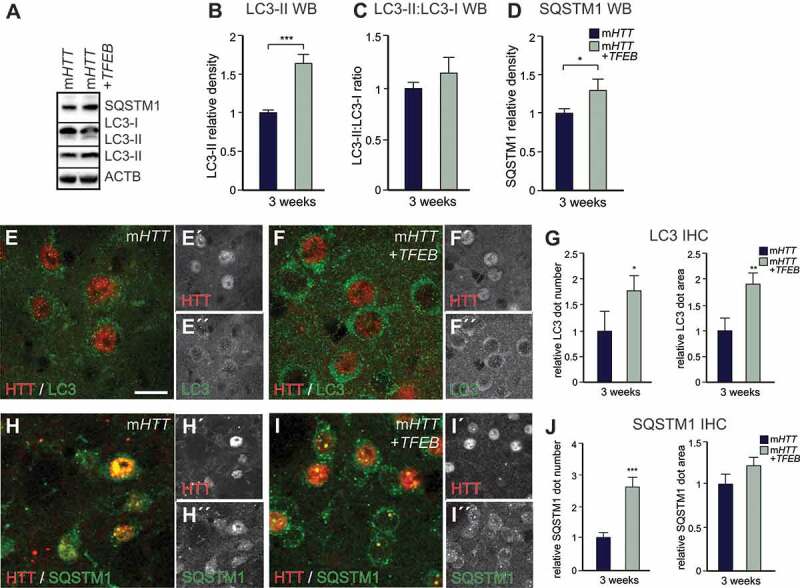
Autophagy induction by human *TFEB* overexpression does not reverse HD-like phenotypes II. (A–D) Increased expression levels of the autophagic marker SQSTM1 and LC3-II are present after AAV-*TFEB* co-injections. LC3-II:LC3-I ratio did not change between groups at 3 weeks post-injection. (n = 8; 4–4 animals in each group). (E–G) LC3 dot number and size were both significantly increased in the co-injected animals compared to AAV-m*HTT*. (n = 12; 3–3 animals in each group). (H–J) The number of SQSTM1 dots significantly increased in the co-injected animals compared to AAV-m*HTT* alone. (n = 10; 3–3 animals in each group). ***p < 0.001; **p < 0.01; *p < 0.05; two-tailed two-sample T-tests were used. All data are shown as mean ± SEM. WB values were normalized to AAV-m*HTT* expression levels and corrected to ACTB values. IHC values were normalized to the AAV-m*HTT* dot number or area. Scale bars: 25 μm

To further verify that *TFEB* overexpression activated autophagy, we used the same panel of autophagic markers for IHC and WB as above (Figure 2 and S1). Animals co-injected with AAV-*TFEB* demonstrated significantly more LC3-II and SQSTM1, while the ratio of LC3-II:LC3-I was not changed (Figure 5). BECN1 levels were similar between m*HTT* and co-injected animals (Fig. S3B-D), while LAMP1 accumulated after *TFEB* overexpression (Fig. S3E-H). These results indicate that while the autophagy process is activated, indicated by the elevated amount of the autophagosome marker LC3-II, it fails to degrade mHTT aggregates. Rather, we detect a clear impairment of autophagy and an accumulation of late autophagic structures.

In the HD models, autophagy impairment results in an increased number of autophagosomes, many of which are empty due to a cargo recognition failure [6]. Our data support this finding since *TFEB* overexpression resulted in increased SQSTM1, LC3-II, and LAMP1 puncta. This result indicates that there is an increase of autophagosomes and lysosomes/endosomes, while cargo is not degraded and mHTT aggregation is not decreased. Altogether, our results suggest that activation of autophagy by *TFEB* is not sufficient to efficiently reduce mHTT aggregation, since it does not address cargo recognition defects and, therefore, results in the accumulation of empty autophagosomes. It is also worth noting that *TFEB* overexpression primarily increased cytoplasmic-localized TFEB. Under normal conditions, TFEB is located in the cytoplasm, but upon stress, starvation, or lysosomal dysfunction, it translocates to the nucleus, where it promotes the transcription of its target genes, resulting in the activation of cellular protein degradation systems [21,35]. The absence of increased TFEB levels in the nucleus may also contribute to the lack of beneficial therapeutic effect that we found upon *TFEB* overexpression.

### Early but not late autophagy induction by Becn1 overexpression enables the reversal of HD-like phenotypes

**Figure 6.**
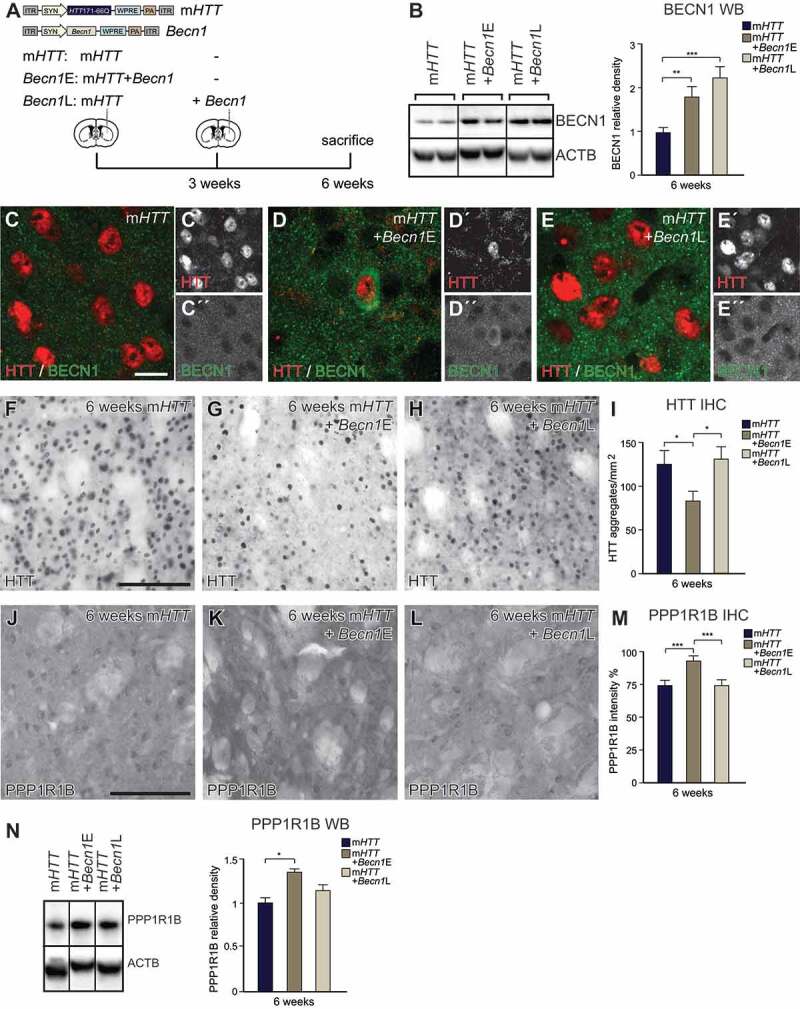
Early but not late autophagy induction by mouse *Becn1* overexpression reverses HD-like phenotypes I. (A) Experimental workflow summarizing the co-delivery of AAV-m*HTT* and AAV-*Becn1* at different time-points. (B–E) WB and IHC analysis verifying successful *Becn1* overexpression after co-injection of m*HTT* and *Becn1* delivered at different time-points. (n = 4; 4–4 animals in each group). (F–I) Co-injection of AAV-*Becn1* and AAV-m*HTT* decreased the aggregation of mHTT but only in the *Becn1*-early group. (n = 20; 4–4 animals in each group). (J–N) PPP1R1B protein levels were significantly higher in the *Becn1*-early group compared to other animals measured with both densitometry and with WB. (n = 20 for IHC; 4–4 animals in each group and n = 4 for WB; 4–4 animals in each group). ***p < 0.001; **p < 0.01; *p < 0.05; One-way ANOVA or nonparametric Kruskal-Wallis test was used depending on normal distribution defined by D’Agostino-Pearson omnibus normality test. All data are shown as mean ± SEM. WB values were normalized to AAV-m*HTT*-injected expression levels and corrected to ACTB values. Densitometry values were normalized to AAV-m*HTT*. Scale bars: 25 μm for C-E and 50 μm for the rest

We and others have previously reported that inducing autophagy by overexpressing *BECN1*, an autophagic regulator that plays a key role in autophagosome formation, mHTT aggregation can be significantly decreased in cell and mouse models of HD, including the AAV-model used in the current study [5,26–28]. On the other hand, mHTT has been reported to increase proteasome-mediated degradation of BECN1 that subsequently impairs autophagy [29]. Thus, the presence of the high levels of mHTT protein or aggregates may impact the therapeutic efficacy of *BECN1*. Therefore, we decided to investigate how disease-stage influences the therapeutic effect of boosting autophagy. We generated an AAV8 vector expressing *Becn1*, as the use of a different AAV serotype for the *Becn1* vector allowed for subsequent AAV-injections without causing an immune response [36]. We designed an experiment consisting of three experimental groups: i) AAV-m*HTT*-only; ii) AAV-m*HTT +* AAV-*Becn1* co-injected together in a naïve mouse brain (m*HTT* + *Becn1*-early) and iii) AAV-m*HTT +* AAV-*Becn1*, where *Becn1* was injected after 3 weeks (m*HTT* + *Becn1*-late), a time-point at which we showed autophagy was already impaired (Figure 6A). All animals were then sacrificed 6 weeks after the start of the experiment.

Successful overexpression of AAV8*-Becn1* was verified using WB and IHC analysis, which demonstrated a significant increase in BECN1 protein levels in both of the co-injected groups when compared to AAV-m*HTT*-injected animals (Figure 6B–Figure 6E). Interestingly, BECN1 showed a drastically increased colocalization with HTT in the m*HTT* + *Becn1*-early group compared to m*HTT*-only and m*HTT* + *Becn1*-late groups (Figure 6C–Figure 6E, S4A, and Table S1).

In order to investigate if *Becn1*-early and *Becn1*-late treatment had beneficial effects on HD-pathology, we first examined the presence of HTT-aggregates using EM48-antibody staining. In the *Becn1*-early group, we found that the number of mHTT aggregates was significantly decreased when compared with the control AAV-m*HTT* injected group, while we did not see this effect in the *Becn1*-late group (Figure 6F–Figure 6I). We measured the density of PPP1R1B fibers by IHC and found a significant increase in the *Becn1*-early group (Figure 6J–Figure 6M and S4B-D). We also measured PPP1R1B-levels in the striatal projection neurons using WB and found almost 50% more PPP1R1B protein in the *Becn1*-early group compared to the AAV-m*HTT*-only group (Figure 6N). To the contrary, in the *Becn1*-late group, we found no evidence for a therapeutic effect as *Becn1*-late expression failed to rescue both mHTT-aggregation and PPP1R1B levels (Figure 6F–Figure 6N and S4B-D). WB analysis confirmed similar overexpression levels of mHTT protein levels in all experimental groups (Fig. S4E). We also verified that the significant decrease in mHTT aggregate number was due to the early *Becn1* administration and not because of the shorter *Becn1* overexpression in the *Becn1*-late group by injecting AAV-m*HTT* + AAV-*Becn1* together and sacrificing animals 3 weeks post-injection (Fig. S4F). We found a significant decrease in mHTT aggregation after 3 weeks of *Becn1* overexpression verifying that only *Becn1*-early but not late overexpression can reverse the HD-like phenotype (Fig. S4 G-I). Together, these data demonstrate that *Becn1* administration is only sufficient for mHTT clearance and PPP1R1B rescue in the *Becn1*-early group.

**Figure 7.**
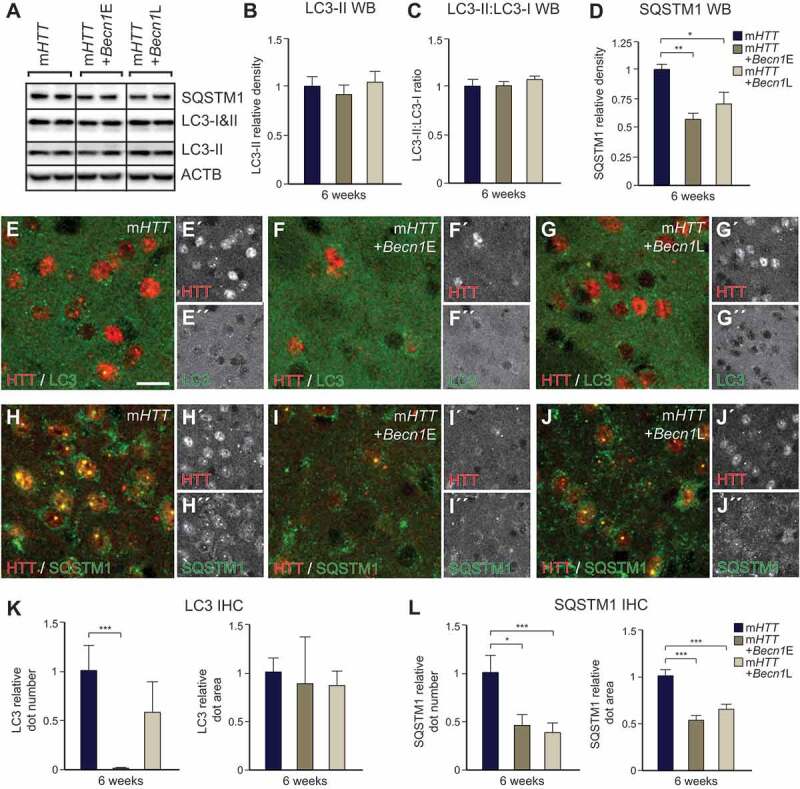
Early but not late autophagy induction by mouse *Becn1* overexpression reverses HD-like phenotypes II. (A–D) Decreased expression levels of the autophagic marker SQSTM1 are present after AAV-*Becn1* co-injections. (n = 6 for m*HTT-*only and *Becn1*-early, n = 9 for *Becn1*-late; 4–4 animals in each group). LC3-II and LC3-II:LC3-I ratio did not change in any of the animal groups at 6 weeks post-injection. (n = 10 for m*HTT*-only and *Becn1*-early, n = 12 for *Becn1*-late; 4–4 animals in each group). (E–L) Both SQSTM1 dot size and number significantly decreased in the *Becn1* co-injected groups compared to the AAV-m*HTT*-only after 6 weeks. (n = 16 for m*HTT*-only and *Becn1*-late, n = 14 for *Becn1*-early; 3–3 animals in each group). LC3 dot number significantly decreased in the *Becn1*-early group compared to the m*HTT*-only, while the area of the LC3 puncta was similar in all groups. (n = 15; 3–3 animals in each group). ***p < 0.001; **p < 0.01; *p < 0.05; One-way ANOVA or nonparametric Kruskal-Wallis test was used depending on normal distribution defined by D’Agostino-Pearson omnibus normality test. All data are shown as mean ± SEM. WB values were normalized to AAV-m*HTT-*injected expression levels and corrected to ACTB values. IHC values were normalized to the AAV-m*HTT* dot number or area. Scale bar: 25 μm

We next investigated how autophagy is altered upon *Becn1* overexpression using WB and IHC. This analysis revealed a significant decrease in SQSTM1 levels in both the *Becn1*-early and *Becn1*-late groups, demonstrating the induction of autophagy in both groups (Figure 7A, Figure 7D, Figure 7H, Figure 7J and Figure 7L). The level of the autophagosomal marker LC3-II was not different in any of the groups using WB analysis, but using IHC, we found a significant decrease in the number of LC3 puncta in the *Becn1*-early group compared to m*HTT*-only (Figure 7A–C, Figure 7E–Figure 7G, and Figure 7K). This reduction in the number of LC3-II-positive puncta correlated with the reduction of the number of mHTT aggregates in the *Becn1*-early group (Figure 7E–Figure 7G, Figure 7K). Interestingly, WB experiments showed a significant increase of TFEB protein level in *Becn1*-late compared to the *Becn1*-early group (Fig. S5A). We found a significant increase of endogenous TFEB puncta by using IHC in the *Becn1*-late group compared to the *Becn1*-early and m*HTT*-only groups (Fig. S5B-E). Additionally, we saw clear reductions in the lysosomal/endosomal LAMP1 dot number, size, and expression levels in the *Becn1*-late group compared to the other groups (Fig. S5F-J). Overall, these data demonstrate a clear induction of autophagy in both the *Becn1*-early and *Becn1*-late groups.

In the *Becn1*-early group, where autophagy was intact, *Becn1* overexpression lowered the amount of mHTT aggregates (Figure 6F–Figure 6I). BECN1 puncta accumulated around mHTT aggregates, resulting in an efficient reduction of mHTT aggregate number (Figure 6D). On the contrary, when *Becn1* was overexpressed at a later time-point, BECN1 puncta showed a diffused staining similar to the m*HTT*-only group (Figure 6C and Figure 6E). Moreover, the level of TFEB (that regulates lysosomal biogenesis and proliferation) increased, while the amount of LAMP1 decreased (Fig. S5). These data indicate that boosting autophagy at a later time-point when prominent autophagy alterations are present, results in a cellular state that does not allow for *Becn1*-mediated degradation of mHTT aggregates. Therefore, early timing of autophagic activation is crucial to enable the reversal of m*HTT* associated phenotypes.

## Discussion

In this study, we used an AAV-model of HD that allows for a temporal investigation of autophagy impairments upon neuronal mHTT aggregation *in vivo*. Initially, m*HTT* expression leads to an enhancement of neuronal autophagy, but this is rapidly replaced with distinct impairments of autophagy during later stages of the disease progression. The alterations in autophagy are a result of both transcriptional and post-transcriptional changes and are characterized by a late autophagy-lysosomal defect. Our results suggest that the presence of mHTT aggregates in striatal neurons results in an initial compensatory response, whereby autophagy is activated in order to remove toxic and aggregated proteins. However, this is rapidly replaced by a decline in autophagy at a later stage, most likely due to increased levels of mHTT aggregation. There are a growing number of studies indicating a complex impairment of autophagy in HD, including several contradictory results [5–8,37-41]. The AAV-based model that we used in this study allows for a detailed and controlled understanding of autophagy impairment in striatal neurons *in vivo* caused by mHTT aggregation. In line with several other studies, we found delayed autophagy impairment coupled with a clear cargo loading and lysosomal transport/fusion failure that correlates with the presence of mHTT aggregates [5,6,8,42]. Our data also supports that complex molecular mechanisms underlie the impairment of autophagy due to m*HTT* expression, including both a transcriptional dysregulation of autophagy gene-networks and effects related to direct protein interactions.

It has previously been suggested that activation of autophagy early in the disease process could be key to successful mHTT aggregate clearance [12,17,43–45]. Chemical screenings for autophagy drug candidates have identified autophagy inducers as HD treatment candidates, such as lithium, trehalose, and rilmenidine [12,46]. These preclinical findings have led to the first open-label study testing rilmenidine, a well-tolerated, safe, centrally acting, anti-hypertensive drug, as a means to upregulate autophagy in mild-severity HD patients [13]. As the disease progresses, the transcriptional and post-transcriptional alterations in autophagy and lysosomal abnormalities become more pronounced, which could hamper autophagy induction therapies. As HD patients can usually be diagnosed before the onset of symptoms, therapeutic approaches to modulate autophagy before the disease onset should be feasible. In addition, it is also becoming increasingly clear that autophagy impairments in different neurodegenerative disorders are distinct and highly disease-specific. In HD, this impairment appears to occur at a late stage in the autophagy pathway, resulting in a buildup of empty autophagosomes. Both of these aspects are important to consider when developing autophagy-activating therapies for neurodegenerative disorders. In this study, we demonstrate clinically relevant scenarios of these challenges.

*TFEB* has been previously shown in mouse and *in vitro* HD models to lower mHTT aggregation, but the level and duration of autophagy activation via this transcription factor needs to be tightly controlled [21,22]. Despite this evidence, we did not find a reduction in mHTT aggregation or restoration in the PPP1R1B levels when we activated autophagy by human *TFEB* overexpression in our AAV-model. While *TFEB* overexpression could increase autophagy, when coupled with increased numbers of autophagosomes and lysosomes, it did not enable the degradation of mHTT aggregates. On the contrary, *TFEB* overexpression led to an accumulation of late autophagic structures and selective autophagic substrates like SQSTM1. This outcome suggests that high levels of *TFEB* expression over time would eventually lead to increased mHTT aggregation and could potentially worsen HD-associated phenotypes. In line with this, inefficient activation of *TFEB* has been suggested to potentially worsen neurodegenerative diseases by increasing the aggregate formation [35].

On the other hand, overexpression of *Becn1* successfully reduced mHTT aggregates in the AAV-model. We found a significant improvement both in the number of mHTT aggregates and in PPP1R1B protein levels. However, this therapeutic efficacy was only found when *Becn1* was administrated early in the disease process. When administering *Becn1* at a later stage, with established mHTT accumulation and autophagy impairments, *Becn1-*mediated autophagy induction did not rescue the m*HTT* associated phenotypes. These observations suggest that the presence of high levels of m*HTT* and mHTT aggregates block BECN1 activity. This result is in line with previous observations that demonstrate HTT protein as a scaffold for selective autophagy where both the HTT and mHTT associates with BECN1 [29,38,47]. WT HTT polyQ tracts are required for protecting BECN1 from proteasome-mediated degradation [29,38,47]. Moreover, WT polyQ tracts bind to BECN1 that enables the deubiquitination and degradation of BECN1, but this interaction is competed by proteins with longer polyQ expansion mutations [29]. Together, these data support a model where *Becn1* activity is blocked in the late-stage disease process due to the presence of high levels of long polyQ tracts that outcompete the WT tracts. Thus, the early activation of *Becn1* is key for therapeutic efficacy.

In summary, our results suggest that while therapeutic strategies boosting autophagy through pharmacological or genetic manipulation are promising, further work is now needed to identify therapeutic targets that could prevent mHTT aggregation and rescue defects in key autophagy steps, like autophagosome transport, vesicle trafficking, fusion to lysosomes, and altered post-translational modification. While initial studies indicate that general autophagy inducers can be beneficial, therapies that correct the specific autophagy-related mechanistic dysfunctions observed in HD may lead to more efficient clearing of mHTT and attenuation of toxicity. Altogether, better understanding of the precise autophagy cargo recognition and transcriptional regulation is key to future therapy development.

## Materials and methods

### Viral vectors

To overexpress *HTT* or m*HTT*, we used AAV vectors encoding a truncated human *Huntingtin* gene with 18 or 66 CAG repeats under the control of a *Syn1* promoter as previously described [5,48]. Truncated *HTT* fragment was 588 base pairs long (~ 21.7 kDa), while m*HTT* fragment was 723 base pairs long (~ 27 kDa). AAV vectors of serotype 5 encoding *HTT* or m*HTT* or mouse *Becn1* or human *TFEB* and serotype 8 encoding mouse *Becn1* were designed and produced as previously described [5]. The titers for AAV5-*HTT*, m*HTT, Becn1* and *TFEB* were: 1.2E+15, 1.5E+15, 6.5E+14 and 9.9E+14 genome copies/ml, respectively. The titer for AAV8-*Becn1* was 2.2E+13 genome copies/ml. The final working dilution in PBS (Thermo Fisher Scientific, 14190094) was 33% for AAV5-*HTT*, m*HTT* and *Becn1*, 50% for AAV8-*Becn1* and 20% for AAV5-*TFEB.*

### Animal surgery

All animal-related procedures were approved and conducted in accordance with the committee for the use of laboratory animals at Lund University. All stereotactic injections into the striatum were performed as described before [5]. All mice were adult C57BL/6 females aged 9 to 10 weeks old at the time of surgery. Unilateral injections were always on the right side of the brain, with a total of 1 µl of the injected virus. The injection coordinates were: AP: +0.9 mm and ML: ±1.8 mm (from Bregma); DV: −2.7 mm (from dura).

### Immunohistochemistry

At 10 d or 3 weeks post-injection all animals were transcardially perfused with ice-cold 4% paraformaldehyde, post-fixed for 4 h in 4% paraformaldehyde, then immersed in 25% sucrose (Merck Millipore, 100892) overnight. Brains were frozen and cut on a microtome in 35 µm sections in series of 5 or 6. Standard IHC was performed on free-floating sections, as published in detail elsewhere [5]. Primary and secondary antibodies were diluted, as summarized in **Table 1**. All fluorescent sections were counterstained with 4ʹ,6-diamidino-2-phenylindole (DAPI, Sigma-Aldrich, 1:1,000). All 3ʹ-diaminobenzidine stained images were taken using a fluorescence microscope (Olympus Life Science, AX70). All fluorescent images were taken using a confocal laser scanning microscope (Leica, TCS SP8).

**Table 1.** List of antibodies used for IHC

Antibody	Source	Species	Dilution	Catalog number	Antibody registry number
BECN1	Santa CruzBiotechnology	Rabbit	1:200	Sc-11427	RRID:AB_2064465
PPP1R1B/DARPP-32	Abcam	Rabbit	1:500	Ab40801	RRID:AB_731843
EM48	Merck Millipore	Mouse	1:200	MAB5374	RRID:AB_177645
LAMP1	Sigma-Aldrich	Rabbit	1:200	L1418	RRID:AB_477157
LC3B	Novus Biologicals	Rabbit	1:500	NB100-2220	RRID:AB_10003146
SQSTM1	Abcam	Mouse	1:500	Ab56416	RRID:AB_945626
TFEB	Bethyl Laboratories	Rabbit	1:100	A303-672A	RRID:AB_11204598
Cy3-AffiniPure donkey IgG	Jackson Laboratory	Mouse	1:200	715-165-151	RRID:AB_2315777
DyLight 488 AffiniPure donkey IgG	Jackson Laboratory	Rabbit	1:200	711-485-152	RRID:AB_2492289
Biotinylated goat IgG	Vector Laboratories	Rabbit	1:400	BA-1000	RRID:AB_2313606
Biotinylated horse IgG	Vector Laboratories	Mouse	1:400	BA-2001	RRID:AB_2336180

### Colocalization analysis

Pictures for colocalization analysis were taken with Leica TCS SP8 confocal laser scanning microscope using the same magnification and settings in 3–3 animals/group. Pictures were analyzed further in Fiji using Just Another Colocalization Plugin (JACoP) (https://imagej.nih.gov/ij/plugins/track/jacop2.html) as previously described [5,49]. Two independent methods were used in order to determine BECN1 colocalization with HTT: Pearson’s coefficient and Li’s intensity correlation. Li’s intensity correlation analysis was also used to define Li’s intensity correlation quotient (ICQ). Colocalization parameters were summarized in **Table S1**. Percentage of colocalization was defined by:

$$\%\;of\;colocalization=Person^{\prime }s\;coefficient\times 100$$

$$\%\;of\;colocalization=Li^{\prime }s\;ICQ\times 2\times 100$$

### Western blot

Injected mice were sacrificed by cervical dislocation and the injected right side of the striatum was dissected out for WB samples. The striatal tissue was lysed and homogenized as described elsewhere [5]. Protein concentration was determined using DC protein assay kit (Bio-Rad, 5000116). 10–15 μg of protein was boiled at 95°C for 5 min in Laemmli buffer (Bio-Rad, 1610737), separated on a 4 – 12% SDS/PAGE gel and then transferred using the Transblot®-Turbo™ Transfer system (Bio-Rad). After 1 h blocking in Tris-buffered saline (TBS; 50 mM Tris-Cl, 150 mM NaCl, pH 7.6) with 0.1% Tween 20 (Sigma-Aldrich, P7949) and 2.5% (wt:vol) nonfat dry milk (Bio-Rad Laboratories‎, 1706404), membranes were incubated overnight at 4°C in one of the primary antibodies summarized in **Table 2**. After washing with TBST, membranes were incubated for 1 h at room temperature with HRP-conjugated secondary antibodies listed in **Table 2**. Protein expression was developed with the ECL™ Prime Western Blotting Detection Reagent (Life Technologies, RPN2232). Signal was captured using a Chemidoc MP system (Bio-Rad). Band intensities were quantified using ImageJ software (ImageJ, 1.48v) by densitometry.

**Table 2.** List of antibodies used for WB

Antibody	Source	Species	Dilution	Catalog number	Antibody registry number
BECN1	Santa CruzBiotechnology	Rabbit	1:1,000	Sc-11427	RRID:AB_2064465
PPP1R1B/DARPP-32	Abcam	Rabbit	1:100,000	Ab40801	RRID:AB_731843
EM48	Merck Millipore	Mouse	1:2,000	MAB5374	RRID:AB_177645
LAMP1 *(Sc)*	Santa CruzBiotechnology	Mouse	1:250	Sc-17768	RRID:AB_626851
LAMP1	Sigma-Aldrich	Rabbit	1:2,000	L1418	RRID:AB_477157
LC3B	Novus Biologicals	Rabbit	1:5,000	NB100-2220	RRID:AB_10003146
SQSTM1	Abcam	Mouse	1:5,000	Ab56416	RRID:AB_945626
TFEB	Bethyl Laboratories	Rabbit	1:1,000	A303-672A	RRID:AB_11204598
HRP-conjugated antibody	Sigma-Aldrich	Rabbit	1:5,000	NA9340	RRID:AB_772191
HRP-conjugated antibody	Santa Cruz Biotechnology	Mouse	1:5,000	Sc-2005	RRID:AB_631736
ACTB (actin beta)	Sigma-Aldrich	Mouse	1:100,000	A3854	RRID:AB_262011

### mRNA sequencing and analysis

The injected right side of the striatum was dissected from decapitated vector-injected mice, frozen on dry ice and homogenized in Tissue LyserLT (50 Hz, 2 × 2 min). RNA was extracted using the miRNeasy mini kit (Qiagen, 217004) and sent for mRNA sequencing to SciLifeLab. cDNA libraries were prepared using the Illumina Strand-specific TruSeq RNA library kit (Illumina, RS-122-2001) using poly-A selection. Illumina high throughput sequencing was applied to all samples.

The 50 base pair single-end reads were mapped to the genome (mm10 for mouse striatal tissue). Reads were quantified to Refseq (mean number of reads mapping to RefSeq annotation: 18,982,016). The normalization and identification of differentially expressed genes was conducted using the Bioconducter/R package DESeq2 [50]. All RNA-seq data analyzed has previously been submitted to NCBI Gene Expression Omnibus (GSE78928). Samples analyzed in this study were: GSM2081317-GSM2081328.

HADb (Human Autophagy-dedicated Database) was used in Figure 3C and Figure 3D [51] and The Human Lysosome Gene Database (hLGDB) was used in Fig. S2E [52].

GO term analysis was performed in PANTHER (http://pantherdb.org/) with GO Ontology database (Release 2018-06-01 [53]). Genes significantly downregulated (p < 0.05, Benjamini-Hochberg corrected) upon m*HTT* 3 weeks versus *HTT* 3 weeks were tested for overrepresentation in PANTHER Pathways [54] and PANTHER GO-SLIM biological process.

### Statistical analysis

For all WB and IHC analysis presented in the main and in the supplementary figures, 10–10 animals were injected in total for each time-point and for each experimental group. Experimental groups were then divided into two subgroups for subsequent WB and IHC analysis (containing 5–5 animals). Inefficient or misplaced viral-injected animals were discarded from further analysis. The number of animals and technical replicates are specified in detail for each experiment in the figure legends.

PPP1R1B densitometry in the striatum was measured on 3 representative sections from each brain using ImageJ. In each case, the non-injected, contralateral site was used for normalization. Amounts of mHTT aggregates were quantified by counting the aggregates in the striatum in 5 representative sections from each brain using ImageJ and then normalized to 1 mm^2^.

Relative SQSTM1, LC3, LAMP1, TFEB, and BECN1 dot number and size were defined with ImageJ as previously described [5]. All pictures taken with the confocal microscopy were done using a magnification of 63x.

Differential expression analysis of RNA-sequencing data was performed using the DESeq2 R package [50], with p < 0.05 (Benjamini-Hochberg corrected) used as a threshold for significance in Wald test.

Two-tailed, two-sample T-tests were used in Figures 1G, Figure 2, Figure 4, Figure 5, S1, S3, and S4I. One-way ANOVA or nonparametric Kruskal-Wallis test was used in Figures 1B, Figures 1H, Figures 6, Figures 7, S4E, and S5 depending on normal distribution defined by the D’Agostino-Pearson omnibus normality test. The criterion for significance for all analysis was p < 0.05. All data are shown as mean ± SEM.

## Supplementary Material

Supplemental Material
