# Supplementary material for: Impact of differential and time-dependent autophagy activation on therapeutic efficacy in a model of Huntington disease
Source: Autophagy. 2020 May 6;17(6):1316–29. doi: 10.1080/15548627.2020.1760014 (PMC8204969; doi:10.1080/15548627.2020.1760014)
Supplement: Supplemental Material [file KAUP_A_1760014_SM7574.docx]

**Figure S1.** m*HTT* overexpression alters autophagy on a protein level in striatal neurons. (**A and B**) Accumulation of LAMP1 was present after both 10 d and 3 weeks in the AAV-m*HTT*-injected, but not in the AAV-*HTT*-injected mice. LAMP1 antibody from Santa Cruz Biotechnology *(Sc)* was used in panel “A” and from Sigma-Aldrich in panel “B” (n = 4; 4 – 4 animals in each group). (**C-F**) While there was a clear increase in LAMP1 expression at 10 d between AAV-*HTT-* and m*HTT*-injected animals, there was no difference between the number of LAMP1 dots at 10 d and 3 weeks. The area of the LAMP1 puncta decreased at later time points in the AAV-m*HTT*-injected animals. (n = 14; 3 - 3 animals in each group). (**G-J**) There was no difference in the TFEB protein level between any groups using IHC (n = 12; 3 - 3 animals in each group) or WB (n = 13; 4 - 4 animals in each group). ***p<0.001; **p<0.01; *p<0.05; two-tailed two-sample variance T-tests were used. All data are shown as mean ± SEM. WB values were normalized to 10 d or 3 weeks AAV-*HTT* expression levels and corrected to ACTB values. IHC values were normalized to 10 d AAV-m*HTT* dot number or area. Scale bar: 25 μm. Related to Figure 2.

**Figure S2.** m*HTT* overexpression alters autophagy on a transcriptional level in striatal neurons. (**A**) PCA dimensionality reduction separates animals injected at 10 d and 3 weeks. (n = 2 for *HTT* and n = 3 for m*HTT* animals/group at 10 d and n = 3 for *HTT* and n = 2 for m*HTT* animals/group at 3-week time-points). (**B**) PCA analysis separates AAV-*HTT* and m*HTT*-injected animals after 3 weeks of injection. (n = 3 for *HTT* and n = 2 for m*HTT* animals/group at 3-week time-points). (**C**) Majority of differentially expressed genes after AAV-m*HTT* injection at 10 d and 3 weeks are significantly decreased. (n = 2 for *HTT* and n = 3 for m*HTT* animals/group at 10 d and n = 3 for *HTT* and n = 2 for m*HTT* animals/group at 3-week time-points). (**D**) Top 9 GO Panther SLIM Biological processes with the highest fold-enrichment among genes significantly downregulated in 3 weeks versus 10 d AAV-m*HTT*-injected animals. Gray bars show fold enrichment. Triangle points show p-value. Numbers indicate the number of significantly downregulated genes for the GO-term. (n = 3 for *HTT* and n = 2 for m*HTT* animals/group at 3-week time-points). (**E**) Heatmap of differentially expressed lysosomal genes show opposite expression levels at 10 d and 3 weeks post-injection. (n = 2 for *HTT* and n = 3 for m*HTT* animals/group at 10 d and n = 3 for *HTT* and n = 2 for m*HTT* animals/group at 3-week time-points). *p< 0.05; Benjamini-Hochberg corrected used as the cutoff for significance in Wald test. Related to Figure 3.

**Figure S3.** Autophagy induction by human *TFEB* overexpression does not reverse HD-like phenotypes. (**A**) Expression level of the truncated HTT was similar between the co-injected animals and AAV-m*HTT*-only. (n = 6 for m*HTT* and 5 for *TFEB* co-injected animals; 4 – 4 animals in each group). (**B-D**) BECN1 levels were not changed in the co-injected animals compared to AAV-m*HTT.* (n = 4; 4 – 4 animals in each group). (**E**) Increased expression level of LAMP1 is present after *TFEB* co-injections. LAMP1 antibody was used from Santa Cruz Biotechnology *(Sc)*. (n = 8; 4 – 4 animals in each group). (**F-H**) The number and size of LAMP1 dots significantly increased in the co-injected animals compared to AAV-m*HTT-*only*.* (n = 15; 3 – 3 animals in each group). ***p<0.001; **p<0.01; *p<0.05; two-tailed two-sample variance T-tests were used. All data are shown as mean ± SEM. WB values were normalized to AAV-m*HTT* expression levels and corrected to ACTB values. IHC values were normalized to the AAV-m*HTT* dot number or area. Scale bar: 25 μm. Related to Figure 4 and Figure 5.

**Figure S4.** Early but not late autophagy induction by mouse *Becn1* overexpression reverses HD-like phenotypes I. (**A**) BECN1 showed a significantly increased colocalization with human HTT in the *Becn1*-early group compared to *Becn1*-late and m*HTT*-only groups. (n = 15 for the m*HTT*-only and *Becn1*-early groups and n = 16 for and *Becn1*-late group; 3 - 3 animals in each group). (**B-D**) Scanned striatal sections stained with PPP1R1B antibody from m*HTT* only and *Becn1* co-injected groups on the right side of the brains. (**E**) HTT expression levels were similar between all groups. (n = 4; 4 – 4 animals in each group). (**F**) Experimental workflow summarizing the co-delivery of AAV-m*HTT* and AAV-*Becn1*. (**G-I**) Co-injection of AAV-*Becn1* and AAV-m*HTT* together decreased the aggregation of human mHTT. (n = 10 for AAV-m*HTT* and n = 15 for AAV-m*HTT* + AAV-*Becn1*; 3 – 3 animals in each group). ***p<0.001; **p<0.01; *p<0.05; One-way ANOVA test was used based on normal distribution defined by D’Agostino-Pearson omnibus normality test in “A” and “E”; two-tailed two-sample variance T-tests was used in “I.” All data are shown as mean ± SEM. Scale bars: 1.5 mm in B for B-D, and 50 μm in G for G and H. Related to Figure 6.

**Figure S5.** Early but not late autophagy induction by mouse *Becn1* overexpression reverses HD-like phenotypes II. (**A**) TFEB expression level significantly differed between the *Becn1*-late and *Becn1*-early group. (n = 4; 4 - 4 animals in each group). (**B-E**) TFEB dot number and area significantly increased in the *Becn1*-late group compared to m*HTT*-only and *Becn1*-early. (n = 12; 3 - 3 animals in each group). (**F-J**) LAMP1 levels decreased in the *Becn1*-late group compared to both the m*HTT*-only and the *Becn1*-early group. LAMP1 antibody was used from Santa Cruz Biotechnology *(Sc)* for the WB experiments. (n = 5; 4 - 4 animals in each group). LAMP1 dot number and size significantly decreased in the *Becn1*-late group compared to both the m*HTT*-only and *Becn1*-early groups. (n = 14; 3 - 3 animals in each group). ***p<0.001; **p<0.01; *p<0.05; One-way ANOVA or nonparametric Kruskal-Wallis test was used depending on normal distribution defined by D’Agostino-Pearson omnibus normality test. All data are shown as mean ± SEM. WB values were normalized to AAV-m*HTT-*injected expression levels and corrected to ACTB values. IHC values were normalized to the AAV-m*HTT* dot number or area. Scale bar: 25 μm. Related to Figure 7.

**Table S1.** Colocalization Parameters.

| **Figure ID** | **Colocalization Parameter** | **Range** | **Value** | **Colocalization %** |
| --- | --- | --- | --- | --- |
| Fig. S4A  m*HTT* 6 w | Pearson’s Coefficient | -1:1 | 0.0179  ±0.0168 | 1.79  ±1.68 |
| Fig. S4A  m*HTT* 6 w | Li’s ICQ | -0.5:0.5 | 0.0255  ±0.0069 | 5.10  ±1.38 |
| Fig. S4A  m*HTT+Becn1*E 6 w | Pearson’s Coefficient | -1:1 | 0.2177  ±0.0227 | 21.77  ±2.27 |
| Fig. S4A  m*HTT*+*Becn1*E 6 w | Li’s ICQ | -0.5:0.5 | 0.0833  ±0.0105 | 16.66  ±2.10 |
| Fig. S4A  m*HTT+Becn1*L 6 w | Pearson’s Coefficient | -1:1 | 0.0586  ±0.0201 | 5.86  ±2.01 |
| Fig. S4A  m*HTT*+*Becn1*L 6 w | Li’s ICQ | -0.5:0.5 | 0.0375  ±0.0069 | 7.50  ±1.38 |
